# A Case of Haematometra Secondary to Cervical Stenosis after Vesicle Vaginal Fistula Surgical Repair

**DOI:** 10.1155/2017/2303840

**Published:** 2017-10-08

**Authors:** Athanase Lilungulu, Willy Mwibea, Mzee Nassoro, Balthazar Gumodoka

**Affiliations:** ^1^Department of Obstetrics and Gynaecology, Dodoma University, College of Health Sciences, P.O. Box 395, Dodoma, Tanzania; ^2^Department of Obstetrics and Gynaecology, Kibaha Clinical Officer Training College, P.O. Box 30282, Coast Regional, Tanzania; ^3^Department of Obstetrics and Gynaecology, Dodoma Regional Referral Hospital, P.O. Box 43, Dodoma, Tanzania; ^4^Department of Obstetrics & Gynaecology, Catholic University of Health and Allied Sciences, Bugando Medical Centre, P.O. Box 1370, Mwanza, Tanzania

## Abstract

*Background*. Haematometra is a rare postobstetrics fistula surgical repair outcome complication; however the condition can be misinterpreted especially in limited resource areas that lack routine ultrasound guidance and with a slowly progressed increase in size of abdomen accompanied with a history of amenorrhoea together with a history of having unprotective sexual intercourse which may increase the possibility of being controversial to full-term gravid uterus. The causes of haematometra might be either due to congenital abnormality of the vaginal canal or acquired iatrogenically. However, any other cause that involved vaginal canal can be a predisposing factor of haematometra. We present a case of a 32-year-old female patient, who had obstetric fistula which was successfully repaired over the past two years. She presented with one-year-and-two-month history of an amenorrhoea that was progressive accompanied with distended abdomen to the extent of looking typically as the gravid uterus. Explorative laparotomy was performed successfully and surgical incision managed by hysterotomy and salpingotomy, whereby approximately ten liters of serosanguinous blood fluid mixed with blood clots was completely suctioned. Despite being a rare condition after vesicle vaginal fistula repair complication outcome, haematometra remains to be relatively common gynaecological condition among female adolescence during postpubertal period.

## 1. Introduction

Haematometra, also known as haematocolpos, is a common gynaecological condition among postpubertal female adolescence caused by congenital abnormality of vaginal canal [1]; however, any other cause involved vaginal canal regardless of the specific age can be a predisposing factor [2]. The causes of haematometra might be due to either congenital abnormality of the vaginal canal or acquired factor [3, 4]. The most lifetime risk factor of haematometra is congenital abnormality of the vaginal canal. It is reported that postpubertal female adolescence having normal secondary sexual characteristics but presented with primary amenorrhoea must be ruled out with the risk of developed haematometra [5].

Haematometra secondary to any gynaecological surgical procedure involving approach through vaginal canal is extremely rare and usually characterized by clinical feature of secondary amenorrhoea [6]. It usually occurs to female of reproductive age group with the specific history of known gynaecological condition involving specific gynaecological surgical procedure [7]. The management of haematometra regardless of the cause is still remaining to be surgical approach through either abdominal or vaginal technique [8]. We report a case of 32-year-old female patient, who presented with one-year-and-two-month history of an amenorrhoea that was progressive accompanied with distended abdomen to the extent of looking typically as the gravid uterus. Explorative laparotomy was performed successfully and surgical incision managed by hysterotomy and salpingotomy, whereby approximately ten liters of serosanguinous blood fluid mixed with blood clots was completely suctioned.

## 2. Case Presentation

**Figure 1:**
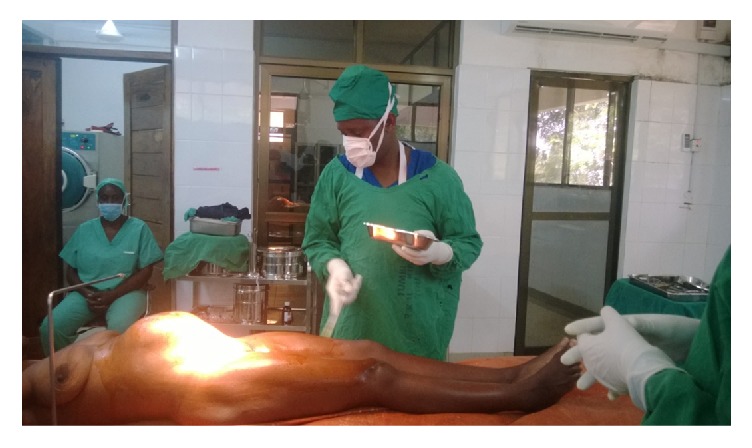
Distended abdomen due to haematometra appears likely to be a full-term pregnancy woman.

A 32-year-old woman, prime para, came at the obstetric department at Kibaha Tumbi Referral Hospital, after an onset of progressive abdomen distention with a history of amenorrhea of one year and two months. Her medical history was unremarkable. However, her past obstetric history was prime para with previous history of prolonged obstructed labor which took three days to be in labor and she had difficult attempted vaginal deliveries of fresh stillbirth male baby at term. On the 14 days after removing the catheter, she started to experience a continuous uncontrolled urine leakage. Before being seen at our obstetric clinic, she had been already having three consecutive obstetric fistula surgical repairs, whereby the third one was done one year and two months prior to this admission. The third fistula surgical repair went successful whereby she could not notice urine leakage and resume her normal sexual life without using any unprotected gear but she could not get a normal menstrual flow throughout the all period, when later she started to notice a progressive abdomen distended and amenorrhea contradict herself as if she had a full-term pregnancy (Figure 1).

Due to the distended abdomen she was thought to have pregnancy despite the fact that she had never seen her normal menstruated blood. Therefore, she was decided to return to the hospital seeking for further evaluation and obstetrics care at our clinic.

Physically she looked healthy with significant normal body weight. Afebrile with her blood pressure measured 120/70 mmHg and pulse rate measured 78 beats/min. Her abdomen was distended with approximated fundal height corresponding to the gestational age of 39 weeks, tense abdomen and tympanic sound, no palpable fetal part, no fluid thrill, and no shifted dullness with the marked tenderness of the lower abdomen.

On speculum examination, a normal cervix could be clearly visualized, and adhesive scared tissue was visualized filling the lateral fornix and the upper part of the vaginal vault. The bimanual examination was performed which revealed that the mass was originated from the uterus with bilateral adnexal difficult to be appreciated.

Haematology laboratory findings revealed a hemoglobin level of 9.6 g/dl, the haematocrit value was 35.7%, and complete blood count was 4.5 billion cells/L with a platelet count of 125 billion/L. Other hematological findings include serum creatinine, BUN, electrolytes, prothrombin time, and partial thromboplastin time appearing in the normal range and biochemistry panel: ALAT-26.71 IU/L ASAT-24.48 IU/L.

Transabdominal ultrasound revealed echogenic opacification with free fluids filled in the uterus, endometrial thickening which measured 10 mm, and markedly thinned elongated closed cervix with free fluid.

The ultrasonography conclusion was a presence of lower genital tract occlusion with haematometra and bilateral haemosalpinx secondarily to acquire obstruction of the lower female genital tract. Detailed examination and planned dilation of the endocervical canal to allow egress of the depicted fluid and subsequent menstruation were attempted under general anesthesia, yet no dimple indicating the external cervical os could be visualized or palpated.

The patient was informed to have explorative laparotomy and the possibility of undergoing abdominal hysterotomy.

**Figure 2:**
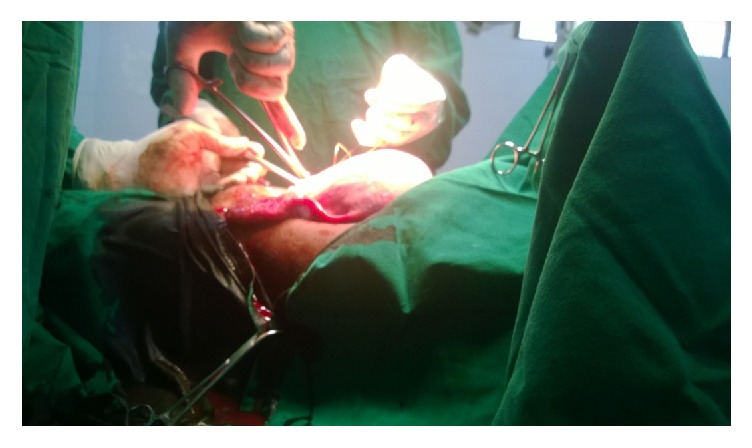
Intraoperative finding of giant haematometra and huge distended bilateral haemosalpinx.

Peritoneal cavity was exposed and found a huge haematometra with distended bilateral haemosalpinx and blood debris leaking at the end of ampulla (Figure 2).

**Figure 3:**
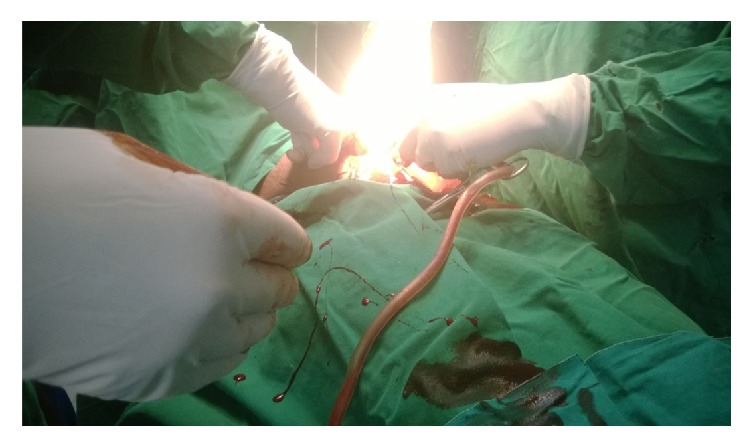
Haematometra blood debris amount measured approximately 10 liters.

The urinary bladder was identified and the lower uterine segment was not obviously formed, hysterotomy was done, and menstruated blood debris was suction which was approximately 10 liters (Figure 3). Using Hegar dilator numbers 6 and 9 repeated alternatively to dilated uterine cervix through abdominally were performed until successful cervical dilatation has been observed and Hegar pass was allowed through the internal os of cervix to the vaginal canal, respectively. Finally, hemostasis was achieved and hysterotomy incision repaired in layers using Vicryl number 2 and abdomen wall closed successfully.

Pathologic assessment of the surgical menstrual blood debris specimen showed the recently evacuated blood clots and endometrial tissues, respectively. The approximate blood loss was one liter and intraoperatively she was transfused one unit of blood. Postoperative period was unremarkable and she was discharged on the seventh day in good condition and advised to continue with cervical dilatation using Hegar at GOPD whereby she was lost to follow-up.

## 3. Discussion

The complications of vaginal occlusion, vaginal stenosis, and vaginal shortening following obstetric fistula surgical repair are almost rare. The risk of complications depends on fistula hole if it has been extended to such an extent that it involved the nearest pelvic organs and presence of postsurgery inflammatory scared band formation [9].

Haematometra defined as the present retention of menstruation cycle blood in the uterine cavity which can be due to congenital or acquired causes. Congenital causes may be due to cervical agenesis, transverse vaginal septum, and imperforate hymen and acquired cause may be iatrogenically as a result of healing by secondary intention with the scared formation [10].

Haematometra has also been described in elderly women secondary to radiotherapy. Cone biopsy can also rarely lead to cervical stenosis followed by haematometra formation.

Cases of haematometra formation after Cesarean section have been reported in various reviewed articles [11].

Haematometra has been reported not only during postabortion evacuation [12], but also during postpartum cervical tear repair, dilatation, and curettage and cervical ablation therapy. Again during caesarian section procedure when attempting to arrest acute uterine blood loss by applying multiple hemostatic sutures material at the placental bed in a case of placenta previa section can lead to synechiae formation, cervical stenosis, and haematometra [13]. Inappropriate closure of anterior and posterior wall of the uterus during lower segment Cesarean scar can create a uterine pouch and lead to haematometra [14].

We report this unusual case of haematometra and bilateral hydrosalpinx due to cervical stenosis after a successful vesicle vaginal fistula surgical repair. In this case, the archived cervical canal obtained successfully by canalizing the cervical internal os by going through the abdominal approach and attempting to do hysterotomy incision whereby an intrauterine cavity dilatation was achieved using different size of Hegar's dilator to allow easy passage and communication between cervical internal os and upper part of the vaginal canal. Later, after cervical os recanalized repair she was stayed in a ward for the fourteen days and then she was discharged based on seeing her in routinely two-week interval for recanalized cervical os by using Hegar dilator. She was again seen at the GOPD clinic in a period of one month after the first surgical repair and found to have being on dilatation schedule which was shown to be successful cervical os canalization. However, she was lost to follow-up to the clinic.

## 4. Conclusion

After surgical fistula repair, cervical stenosis causing haematometra is an uncommon complication diagnosis that can be made in a case of prior cervical surgeries.
